# Intradermal Lactate Monitoring Based on a Microneedle Sensor Patch for Enhanced In Vivo Accuracy

**DOI:** 10.1021/acssensors.4c00337

**Published:** 2024-05-23

**Authors:** Qianyu Wang, Águeda Molinero-Fernandez, Qikun Wei, Xing Xuan, Åsa Konradsson-Geuken, María Cuartero, Gastón A. Crespo

**Affiliations:** † Department of Chemistry, KTH Royal Institute of Technology, Teknikringen 30, SE-114 28 Stockholm, Sweden; ‡ UCAM-SENS, Universidad Católica San Antonio de Murcia, UCAM HiTech, Avda. Andres Hernandez Ros 1, 30107 Murcia, Spain; § Section of Neuropharmacology and Addiction Research, Department of Pharmaceutical Biosciences, Uppsala University, SE-751 05 Uppsala, Sweden

**Keywords:** wearable sensor, interstitial fluid lactate, electrochemical sensor, intradermal measurement, wearable validation

## Abstract

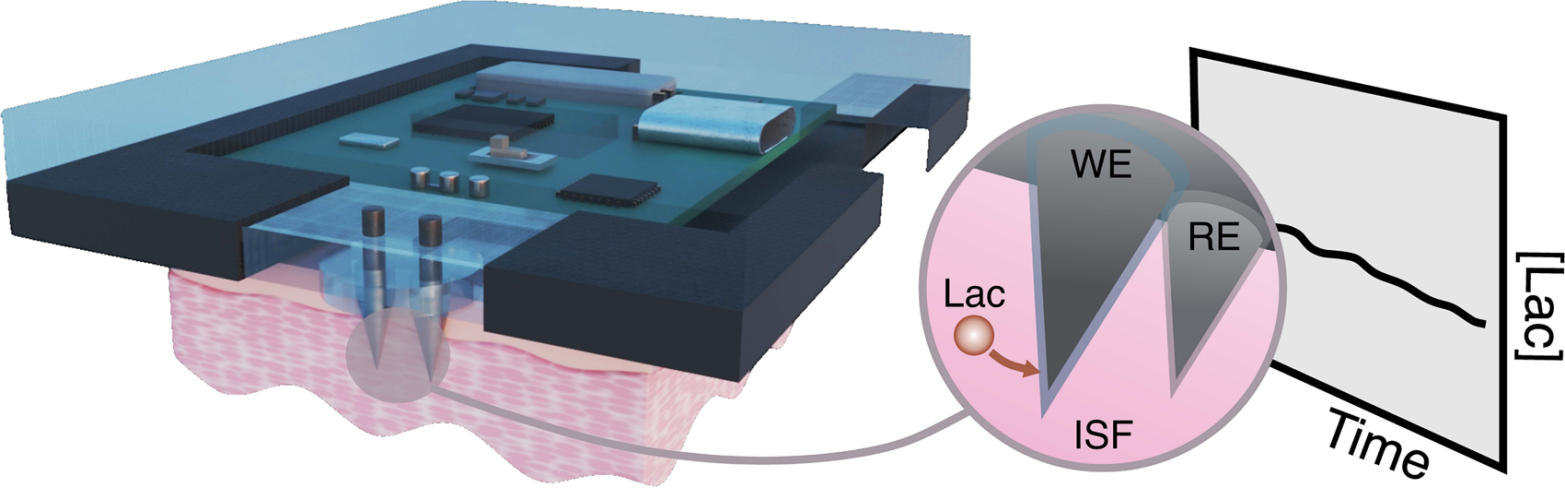

Lactate is an important diagnostic and prognostic biomarker of several human pathological conditions, such as sepsis, malaria, and dengue fever. Unfortunately, due to the lack of reliable analytical decentralized platforms, the determination of lactate yet relies on discrete blood-based assays, which are invasive and inefficient and may cause tension and pain in the patient. Herein, we demonstrate the potential of a fully integrated microneedle (MN) sensing system for the minimally invasive transdermal detection of lactate in an interstitial fluid (ISF). The originality of this analytical technology relies on: (i) a strategy to provide a uniform coating of a doped polymer-based membrane as a diffusion-limiting layer on the MN structure, optimized to perform full-range lactate detection in the ISF (linear range of response: 0.25–35 mM, 30 s assay time, 8 h operation), (ii) double validation of ex vivo and in vivo results based on ISF and blood measurements in rats, (iii) monitoring of lactate level fluctuations under the administration of anesthesia to mimic bedside clinical scenarios, and (iv) in-house design and fabrication of a fully integrated and portable sensing device in the form of a wearable patch including a custom application and user-friendly interface in a smartphone for the rapid, routine, continuous, and real-time lactate monitoring. The main analytical merits of the lactate MN sensor include appropriate selectivity, reversibility, stability, and durability by using a two-electrode amperometric readout. The ex-vivo testing of the MN patch of preconditioned rat skin pieces and euthanized rats successfully demonstrated the accuracy in measuring lactate levels. The in vivo measurements suggested the existence of a positive correlation between ISF and blood lactate when a lag time of 10 min is considered (Pearson’s coefficient = 0.85, mean difference = 0.08 mM). The developed MN-based platform offers distinct advantages over noncontinuous blood sampling in a wide range of contexts, especially where access to laboratory services is limited or blood sampling is not suitable. Implementation of the wearable patch in healthcare could envision personalized medicine in a variety of clinical settings.

Lactate (Lac) is a byproduct of glucose metabolism, and its main generation pathway depends on glycolysis.^1^ The use of blood Lac as a biomarker has been extensively proposed for screening, risk stratification, and prognostication in a wide spectrum of clinical cases.^2^ To illustrate, knowing Lac levels and fluctuations over time is essential in the management of infection-related symptoms in illnesses such as sepsis, malaria, and dengue fever, as well as in noninfectious disorders, including heart failure, trauma, and surgery.^1,2^ In such a context, blood Lac concentrations are commonly assessed in venous blood samples that are analyzed in central laboratories.^3^ This procedure requires trained personnel, expensive equipment, and overhead, and has a turnaround time of up to several hours. With the arrival of new bedside approaches, also called point-of-care testing (POCT), the response-action time has drastically been reduced. However, the current POCT for Lac still requires blood sampling, which is inherently invasive and provides only discrete measurements over time. Moreover, both venous and arterial punctures are uncomfortable and can lead to stress and complications, while the poor concordance of capillary with arterial lactate restricts its use in clinical settings.^3,4^

A noninvasive, user-friendly, and reliable wearable Lac sensor available for POCT would revolutionize the way of managing certain diseases and providing treatment. In principle, to be compatible with this philosophy, it seems better to target biofluids that are more peripheral than blood. Interstitial fluid (ISF) is indeed a logical alternative since its composition is known to be correlated with that of blood.^5^ Effectively, ions and small molecules, such as glucose and lactate, can freely diffuse from blood vessels to ISF via capillaries.^6,7^ In addition, ISF presents a rather stable biological environment (e.g., pH, temperature, and flow velocity), and microneedle-based (MN) sensors are able to penetrate the skin and detect important biomarkers in it, avoiding relevant pain and discomfort.^8,9^ Consequently, the past few years have seen a surge in the development of MN technology, e.g., continuous glucose monitoring systems.^10,11^

Some Lac MN sensors have already appeared in the literature, with the electrochemical readout being the most utilized. Table S1 in the Supporting Information summarizes the most interesting characteristics of those reported platforms.^12−23^ Chronoamperometry is the technique that stands for the merits of high sensitivity, wide detection range, fast response, and seamless integration into portable devices.^21,22^ However, to the best of our knowledge, none of the reported devices have demonstrated the analytical maturity and technological readiness for reliable application in bedside clinical environments. Notably, the detection limits (LODs) of these MN sensors are often low enough to cover clinical measurements of basal Lac (0.3–2.5 mM) but may not be able to accurately measure higher concentrations, such as those expected in certain illnesses as well as dehydration and exhaustion conditions (5–25 mM).^12−15,18,19,24^ In some instances, the diffusion-limiting layer incorporated between the Lac sensing element and the sample was not sufficient to extend the dynamic range of response up to the desired levels.^14,15,25−27^ Furthermore, most of the reports about MN sensors for Lac detection tend to advance their development only until the in vitro stage.^12−17,20^ Then, in cases showing in vivo experiments, the validation of such on-body measurements is normally underestimated. Instead of using collected ISF, the blood-ISF Lac correlation is considered for validation. However, blood-ISF correlation has not yet been fully demonstrated for all of the possible ions and molecules. For Lac, recent studies have shown that concentrations in blood and ISF do not always correlate well, presenting a lag time and varying with certain inducers (e.g., anesthesia conditions).^28,29^ Without a proper validation strategy leading to accuracy evaluation, the analytical potential of new devices can be wrongly overestimated.

In this work, we report a new MN sensor patch for Lac determination in ISF, demonstrating its analytical operation and accuracy following in vitro, ex vivo, and in vivo experiments. In addition, two validation procedures are explored for accuracy evaluation: (i) through ISF collection and analysis and (ii) using blood levels. Importantly, MNs are conceived as a minimally invasive approach based on transdermal measurements. To achieve the goals of real-time monitoring while being compatible with POCT criteria, the MN device incorporates fully integrated electronics comprising an amperometry board for data acquisition and wireless data transmission together with a custom application for data visualization (including the performing of a calibration graph and profile conversion from current to dynamic concentration). The ensuring of the linear range of response (LRR) and response time covering clinical expectations was achieved by incorporating a doped polymer as an outer diffusion layer. The proven analytical characteristics and robustness of the developed MN system allowed us to perform pioneering studies about the relationship between blood and ISF Lac in rats, additionally mimicking a clinical scenario involving anesthesia. Importantly, a double validation protocol considering blood and ISF extraction is implemented for accuracy evaluation. The strong agreement between Lac concentrations measured by the MN sensor and the reference methods revealed that the developed analytical platform is a promising, robust, and accurate tool for further use in diverse clinical settings.

## Experimental Section

### Lac MN Sensing Device

**Figure 1:**
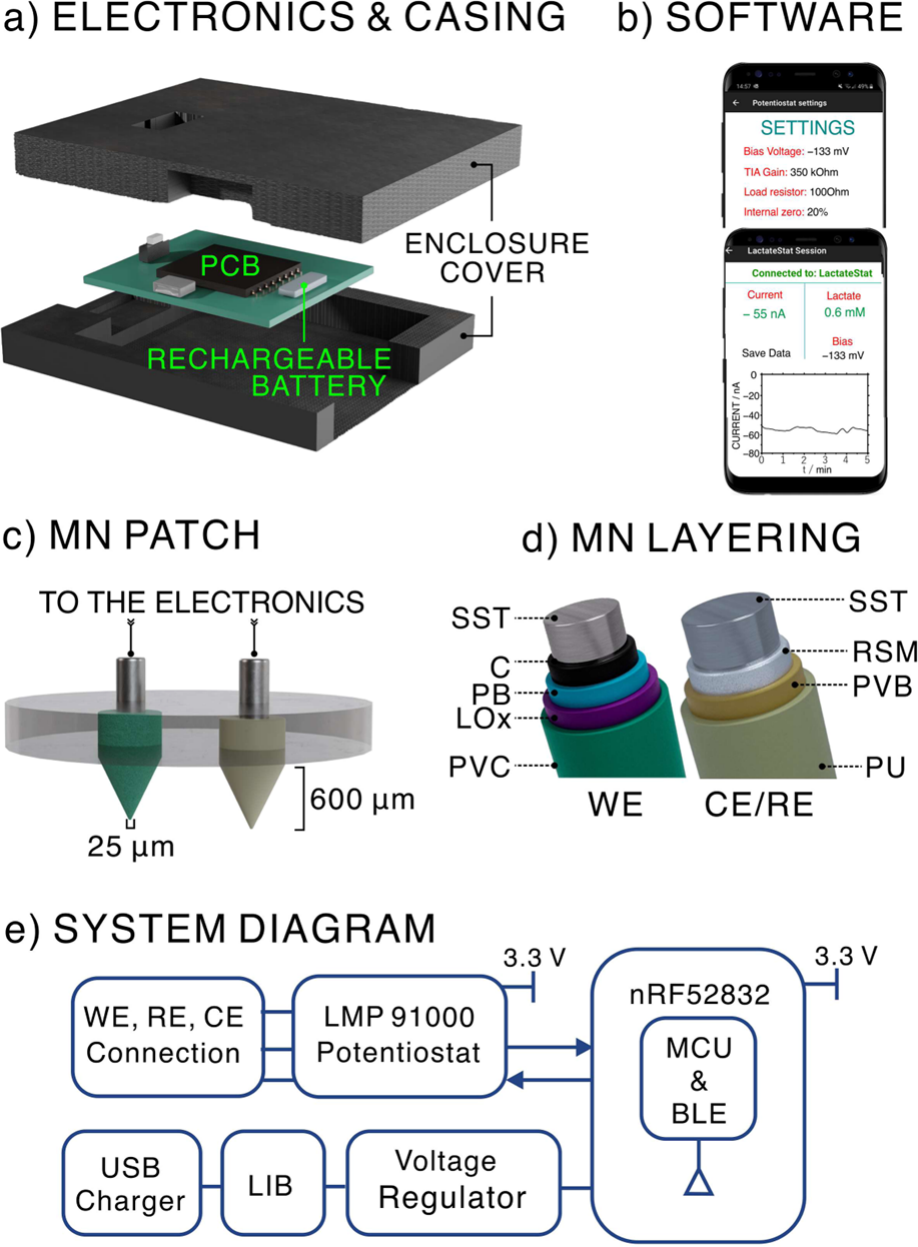
(a) Components and assembly of the electronics and casing. (b) Control panel interface for settings and real-time signal recordings. (c) MN sensor patch. (d) Layer-by-layer composition of the two MNs: WE and CE/RE. (e) Illustration of the system-level block diagram.

The Lac MN sensing device consisted of a reusable electronic board previously developed by our group for signal (current) recording and wireless data transmission (Figure 1a),^30^ custom mobile application with a user-friendly control panel interface (Figure 1b), and a core sensor patch based on two MNs fixed in a flexible silicone rubber-based substrate (Figure 1c,d). The application provides the user with control over the applied voltage, gain, and load resistors for resolution, range of signal output, real-time signals disapply, and save data function. More details on the device elements are provided in Figures S1–S3 in the Supporting Information. A 3D-printed enclosure cover was used to protect the electronics, performing the connection to the MN sensors through it (Figure 1a). The systematic functional block diagram of the device is displayed in Figure 1e. More details on materials and fabrication procedures are provided in the Supporting Information.

The MN sensor patch comprised a two-electrode system [working electrode (WE) and counter-reference electrodes (CE/RE)] to perform amperometric measurements (i.e., dynamic current readout at a constant applied potential). The detailed layer-by-layer construction of the WE and CE/RE is illustrated in Figure 1d. For the WE, a first carbon layer (C-MN) was added to the stainless steel solid MN, and then a layer of Prussian Blue (PB) was electrodeposited by running 20 cyclic voltammetry cycles from −0.5 to 0.6 at 0.05 V s^–1^ in a solution comprising 2.5 mM FeCl_3_, 2.5 mM K_3_[Fe(CN)_6_], 100 mM KCl, and 100 mM HCl. Prior to curing in the oven (100 °C, 1 h), the PB-C-MNs were cleaned in 100 mM HCl to eliminate any residual solution or unattached PB coming from the electrodeposition process.

Next, a mixture of chitosan and lactate oxidase enzyme (CHI-LOx, 1:1 v/v ratio) was prepared by mixing 30 mg/mL LOx (dissolved in 0.1 M phosphate buffer, pH = 7.5) with 1% wt. CHI (dissolved in 0.8 wt % acetic acid). Then, four layers (each with a volume of 0.5 μL) of the CHI-LOx mixture were drop-casted onto the PB-C-MN surface. Each layer was allowed to dry at room temperature for 20 min. Afterward, a volume of 1 μL of a solution of polyvinyl chloride (PVC) doped in 9 wt % with tetradodecylammonium tetrakis(4-chlorophenyl)borate (ETH 500) in tetrahydrofuran (THF) was drop-casted three times. Finally, the WE MN was stored in a phosphate buffered saline solution (PBS, 0.01 M, pH = 7.43) at 4 °C before its usage. Regarding the CE/RE, this was prepared as reported elsewhere.^30^ Briefly, three layers of 1 μL of a solution containing 78 mg of poly(vinyl butyral) (PVB) and 50 mg of NaCl (in 1 mL methanol) were drop-casted onto an MN with an Ag/AgCl coating. The MN was then conditioned overnight in 3 M KCl, dried at room temperature, and an outer polyurethane layer of 1 μL (PU, 20 mg in 1 mL of THF) drop-casted one time was added. The CE/RE MN was stored in 3 M KCl before usage.

## Results and Discussion

### In Vitro Characterization of the MN Sensor Patch for Lac Measurements

**Figure 2:**
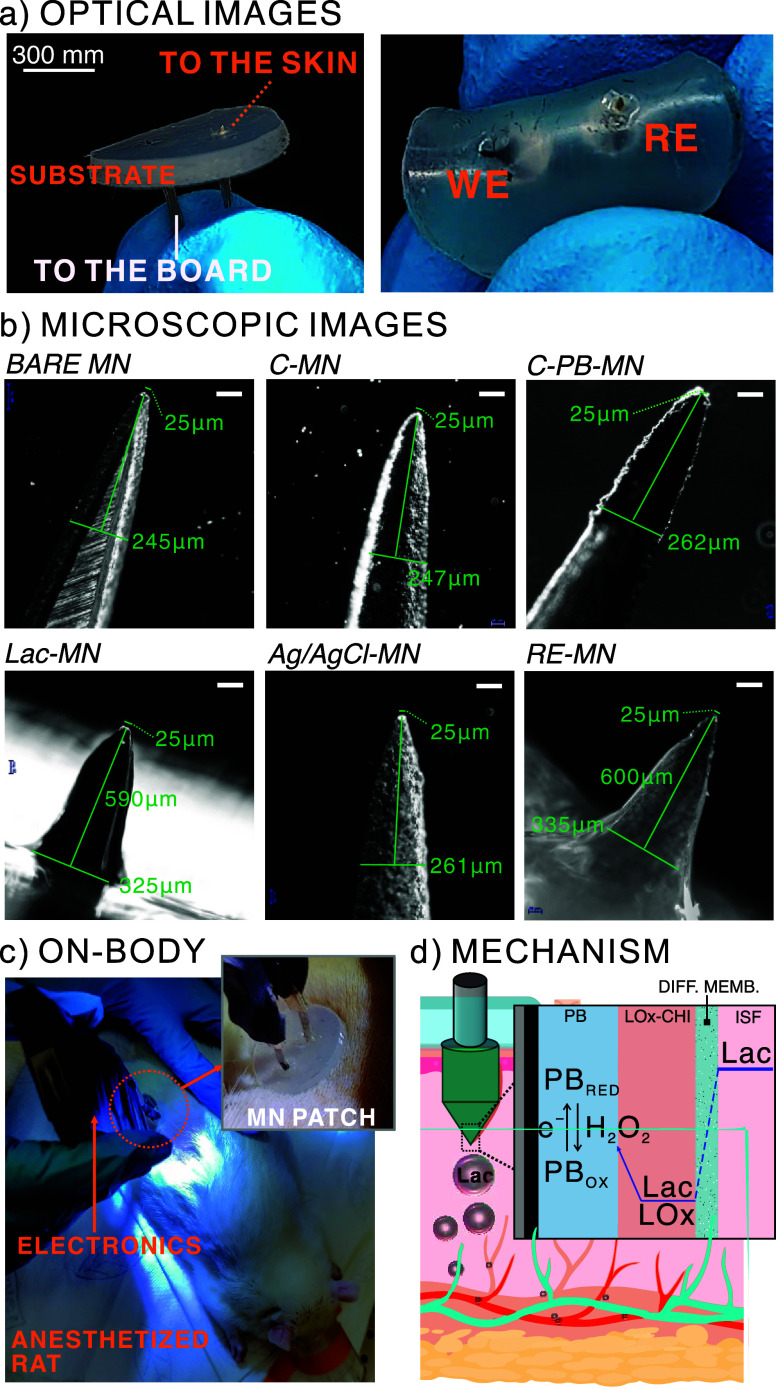
(a) Optical images of the MN patch. (b) Optical microscopic images of individual MNs along the modification process. Scale bar = 100 μm. (c) Image of on-body measurements on anesthetized rats. (d) Working mechanism for Lac detection in ISF. PB stands for PB. RED = reduced. OX = oxidized. LOx = enzyme. LOx-CHI is the enzyme entrapped in the chitosan matrix. DIFF. MEMB. = diffusion membrane. ISF = interstitial fluid. Lac = lactate.

Figure 2a displays real images of the MN sensor patch, i.e., the WE and CE/RE MNs implemented into the substrate. The MN sensor patch was designed to possess high mechanical flexibility and physical stability. The use of stainless steel as the MN core material provides high mechanical strength and adhesion compatibility with the layers required for MN modification. On the other hand, the silicone rubber as the patch material offers adequate flexibility and movement adaptability while keeping the MNs perfectly fitted to it. Then, we characterized the MN dimensions by means of images taken with an inverted optical microscope (Nikon Eclipse Ti2). The results are shown in Figure 2b. After modification, both the WE and RE MNs presented an insertion length of 600 μm, base diameter < 350 μm, tip diameter < 25 μm, and tip angle of 30°. In principle, these dimensions are in agreement with a nonpainless insertion into the skin.^6^ Further SEM images (Apreo 2, Thermo Fisher Scientific) show that the MN sensors maintain their morphology without deterioration of the sensing elements before and after insertion into the rat skins (Figure S4). A careful visual inspection confirmed the integrity of the tip coating membrane with no signs of degradation or detachment. Figure 2c displays an image of the device when on-body measurements were performed in anesthetized rats. Of note, the MN patch was manually applied for MN insertion into the skin.

The required penetration force of the MN sensor patch was evaluated by using a texture analyzer (CT3, VWR) (Figure S5a). The MN sensor showed 0.29 ± 0.03 N of peak force during penetration into the skin mimicking hydrogels,^31^ and 3.2 ± 0.5 N into rat skins (*n* = 5). The ideal force range is suitable for a convenient manual application of the MN sensor patch to the skin with proper adhesion. In addition, Figure S5b shows the microscopic images of both the WE and RE MNs before and after insertions into hydrogels and rat skins. Again, we found that the MNs still maintained an intact morphology without damage or significant film removal after the insertion force test. Notably, rat skins possess a degree of viscoelasticity and elasticity and a more complicated structure.^32,33^ It is therefore essential to render the rat skin planar and smooth for precise measurement.

The penetration depth of the MN was measured using hydrogels since they offer a convenient method for initial evaluation. As shown in Figure S6, microscopic images revealed that the shape of MNs can be clearly preserved by using the hydrogel, and thus, the penetration depth can be accurately measured. In this regard, the penetration depths were measured to be 591 and 606 μm for the Lac MN and RE MN, respectively. The data match the designed length of the MN which was around 600 μm.

The working principle underlying the Lac sensing is provided in Figure 2d. In essence, it is based on the first generation concept, which is achieved through the following elements (from the inside to outside the MN structure): (i) a confined layer of the redox mediator, in this case PB; (ii) the immobilized enzyme (LOx) that is involved in the production of hydrogen peroxide (H_2_O_2_) upon reaction with Lac; and (iii) an external membrane comprising plasticized PVC that controls the amount of lactate reaching the enzyme. This latter element is a diffusion-limiting layer that allowed us to tune the LRR of the WE, which was adjusted to the Lac levels expected in ISF.^21,34,35^ Once Lac crosses the diffusion layer, it interacts with the LOx, generating hydrogen peroxide (H_2_O_2_). Then, the H_2_O_2_ is spontaneously reduced by the PB, the oxidized form of which is activated by applying a constant potential of −0.1 V. This is translated into a change in the Faradaic current of the system. Changes in the Lac concentration that are involved in the H_2_O_2_ formation will generate a correlated change in the current, indirectly allowing for the quantitative determination of Lac.

**Figure 3:**
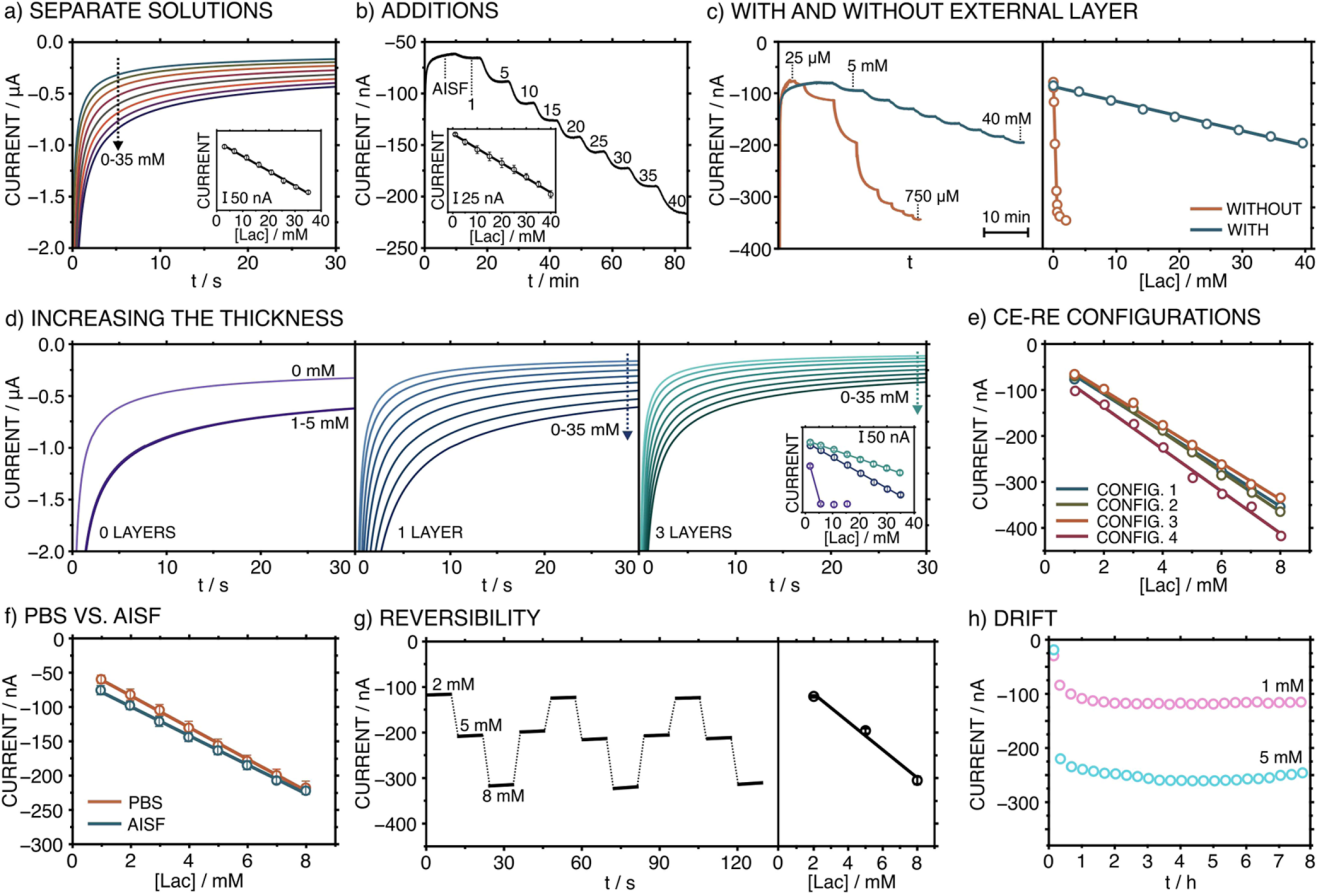
In vitro characterization. (a) Current response for separate solutions containing increasing Lac concentration in the AISF background. Inset: the corresponding calibration graph. (b) Dynamic current response toward increasing Lac concentrations achieved by additions to the stirred AISF background. Inset: the corresponding calibration graph. (c) Left: Dynamic current responses were observed with Lac MNs without (orange) or with (blue) the outer layer. Right: the corresponding calibration graphs. (d) Response of MN sensors prepared with 0, 1, and 3 layers of the outer layer toward increasing Lac concentrations in separate solutions. Inset: the corresponding calibration graphs. (e) Calibration graphs of the same WE MN operated under different arrangements for RE and CE. Configuration 1: WE vs commercial RE and CE. Configuration 2: WE vs MN RE and commercial CE. Configuration 3: WE vs pseudocommercial CE/RE. Configuration 4: WE vs pseudo-MN CE/RE. (f) Calibration graphs obtained in PBS and AISF backgrounds. (g) Left: Reversibility study. Sequence for the Lac concentrations: 2 → 5 → 8 → 5 → 2 → 5 → 8 → 5 → 2 → 5 → 8 mM. Right: the averaged calibration graph. (h) Stability test in solutions containing 1 and 5 mM Lac concentrations in AISF.

The analytical figures of merit of the MN-based Lac sensor were obtained via a series of experiments in a beaker configuration. The entire patch (WE MN and CE/RE MN) was used in this study. We performed two different protocols to evaluate the response toward increasing Lac concentrations. The first protocol was based on the current measurement at a fixed potential (applied potential of −0.1 V) and time (30 s) of separate Lac solutions. The second protocol was based on the dynamic recording of the current by continuously adding Lac to a stirred solution, while the electrode was activated at an applied potential of −0.1 V. Figure 3a,b show the dynamic current and the corresponding calibration graphs in the artificial interstitial fluid (AISF) background. With the first protocol, Lac MN exhibited a LRR from 1 to 35 mM, sensitivity of −8.04 nA mM^–1^, and limit of detection (LOD) of 0.0148 mM (S/N = 3 criteria). A steady-state current was attained within 30 s, with this value being maintained for longer periods (e.g., up to 240 s, see Figure S7). Accordingly, the calibration is valid for continued measurements beyond 30 s. With the second protocol, a rather similar LRR (1–40 mM) was obtained, but with a lower sensitivity (−3.59 nA mM^–1^) and a higher LOD (0.437 mM). The differences between the two methods are likely due to the different diffusion regimes in the sample, since in the second protocol, the sample is under stirring. Despite presenting lower sensitivity, it seems more appropriate to use the first protocol for the calibration in any on-body measurements, because the ISF flow is so low as to be considered a close-to-static condition.

Advantageously, regardless of the calibration method, the LRR was found to include Lac levels expected in ISF. In healthy individuals at rest, Lac levels were reported to be around 0.3–2.5 mM in blood and ISF.^3^ However, the Lac level may increase as a consequence of some clinical conditions, such as sepsis.^36^ Variations from 2 to 5 mM and above 5 mM have been reported for hyperlactatemia and lactic acidosis cases.^36^ Therefore, a sensor to be used in clinical settings must have a minimum linear range of ca. 0.5–5 mM. In contrast, when practicing a physical activity (e.g., sports), higher Lac levels are expected, and hence, the upper limit of the LRR needs to be higher. For example, during intense anaerobic exercise, the level may rise to 15 mM or even higher. Taking all this into account, to provide the sensor with the most versatility, it would be convenient to have a wide LRR. The MN-based Lac sensor encompasses concentrations from 0.5 to 25 mM, making it useful not only for clinical applications but also for sport science, among others.

As reported by our group,^29^ the external plasticized polymeric layer (see composition in the Experimental Section) covering the MN has indeed the capacity to tune the LRR. As observed in Figure 3c, a wider LRR was obtained in the presence of such a layer (0.25–35 mM versus 0.1–0.75 mM with and without the outer layer). Moreover, it is possible to control the LRR, but at the expense of sensitivity. Figure 3d displays the current responses for MNs prepared with 0, 1, and 3 layers of 1 μL-volume of the outer film, indicating an increasing thickness. Similar LRRs (0.25–35 mM) were obtained for 1 and 3 layers, with a decrease in sensitivity (−13.9 vs −6.9 nA mM^–1^, for 1 and 3 layers, respectively) and rather similar repeatability (RSD for the slopes of 6 vs 4% for three equal MNs).

Instead of a three-electrode configuration (WE, RE, and CE), the proposed patch uses a two-electrode configuration based on the use of a pseudo-CE/RE MN. Figure 3e shows the calibration graphs obtained by using different electrode arrangements for the RE and CE elements: commercial RE (single junction Ag/AgCl) and CE (Pt rod), CE and RE MN, pseudo-CE/RE based on the commercial single junction Ag/AgCl electrode, and only using the pseudo-MN CE/RE. This study encompassed the transition from a conventional three-electrode system to a more streamlined two-electrode system with the substitution of commercially available electrodes with the CE/RE MN. The LRR and slope were maintained regardless of the nature, number, and type of electrodes used: (i) commercial Ag/AgCl RE and commercial Pt CE (COMM-RE); (ii) commercial Ag/AgCl RE acting as a C/RE; (iii) Ag/AgCl MN as the RE and commercial Pt as the CE; and (iv) the Ag/AgCl MN as the C/RE (see Table S2 for the calibration parameters). Indeed, the variations found in the slope were smaller than those displayed in the reproducibility tests (6 vs 9%, see below). Accordingly, the two-electrode configuration was confirmed to be suitable for the Lac measurements. This arrangement has the advantage of reducing the complexity of the patch, with a lower number of MNs producing less discomfort in the patient.^6^

In principle, the pseudo-CE/RE MN configuration is expected to support the necessary current magnitude in our experiments (<1 μA) while ensuring a constant potential (i.e., minimal risk of inducing elemental changes on the Ag/AgCl element).^37^ Moreover, the developed CE/RE MN demonstrated a high capacity for ion expelling: it successfully maintained a constant electromotive force (EMF) in solutions containing 10^–5^–10^–1^ M concentration of chloride ions, in contrast to the response presented by an Ag/AgCl MN (Figure S8a). Remarkably, the long-term stability of the RE MN was verified in AISF by comparing the offset of the EMF with that of the commercial double-junction Ag/AgCl RE (drift of 0.04 mV h^–1^, Figure S8b).

To evaluate the selectivity, the effect of the main possible interferences on the amperometric signal was investigated. The presence of glucose, pyruvate, urea, and ascorbic acid did not influence the current response of 1 mM Lac (Figure S9 in the Supporting Information). However, the calibration graph of the Lac MN patch was found to present a slightly lower sensitivity when the background was AISF than that in PBS (Figure 3f): (−21.0 vs −23.8 nA mM^–1^, 9%RSD). According to this matrix effect, it is advisible to perform the calibration in the ASIF medium, which emulates the real intradermal environment where the on-body measurements will be carried out.

The repeatability was evaluated with triplicate consecutive measurements of solutions containing 1, 2, 5, and 8 mM of Lac using the same MN patch and then averaging the calibration graph (Figure S10). Acceptable variations were observed for the respective currents (2, 5, 5, and 1% for the RSDs) but also in the slope and intercept of the calibration (−30.72 ± 0.32 nA mM^–1^, and −93.27 ± 4.84 nA). The reproducibility between electrodes was studied with three electrodes. The slope showed an RSD of 9% (−31.55 ± 2.89 nA mM^–1^) and the intercept had an RSD of 20% (−136.27 ± 27.09 nA). Accordingly, it is convenient to calibrate any MN before being used because, as expected, these will present slightly different calibration parameters between them.

The reversibility was assessed by measuring separate solutions containing increasing and decreasing Lac concentrations (from 2 to 8 mM) during 3 cycles. The current traces observed during the last 5 s of the 30 s measurements, together with the averaged calibration graph, are displayed in Figure 3g. Acceptable variations were observed for the respective currents (3, 5, and 5% for the RSDs of 2, 5, and 8 mM Lac concentrations), but also for the slopes and intercepts in the calibration graphs (−31.74 ± 2.14 nA mM^–1^ and −52.49 ± 8.69 nA). The long-term stability of the Lac MN sensor patch was first conducted in AISF with 1 mM Lac (e.g., physiological Lac concentrations), recording the signal every 30 min (Figure S11). As observed, the sensor can maintain excellent stability with minimal drift during the first 9 h (max. 0.30 nA h^–1^) and displayed a gradual decrement of the signal within the following 2 days. This recommends a daily replacement, making the sensor disposable. To further confirm the drift of the MN sensor, the medium-term stability was characterized in concentrations of 1 and 5 mM for an 8 h observation period at time intervals of 20 min (Figure 3h), showing drifts of 0.29 and 1.41 nA h^–1^, respectively.

Finally, the MN patch was coupled with the portable electronics, reaching the final configuration (Figure 1). The sensing performance of the wireless wearable system was compared with the laboratory potentiostat workstation (Autolab, Metrohm Nordic AB, Sweden) to ensure the accuracy of the measurements. No significant differences were obtained between the slope and intercept of the calibration shown by the lactate MN using both systems (<10% of variation in the slope and intercept using the same electrode, which is indeed within the range observed for repeatability, see above).

### Ex Vivo Characterization of the MN Sensor Patch for Lac Measurements

Both the resiliency of the MN sensor patch to skin insertion and the accuracy of intradermal Lac measurements were demonstrated with ex vivo tests. We considered two approaches: (i) the determination of Lac in pieces of rat skin overnight conditioned in Lac, and (ii) the determination of Lac in euthanized rats. In both cases, a double validation was accomplished, measuring the Lac content in collected ISF samples with Lactate Scout and ion chromatography (IC).

First, the resiliency of the MN patch to skin insertions was investigated by recording the calibration graphs for Lac in AISF before and after skin penetration (Figure S12). Similar slopes (RSD = 2%) and intercepts (RSD = 3%) were obtained. However, higher variations were observed after three insertions. Accordingly, if more than two insertions are performed with the same MN patch, a postcalibration is needed to avoid inaccurate results. Figure S13a shows images of the vertical manual insertion of the MN system into a rat skin sample. The MNs exhibited smooth entry with the penetration length being dictated by the length of the needle. Visual inspection of the site after MN insertion revealed a microhole pattern, with a diameter of approximately 320 μm, which agrees with the base of the MNs (Figure S13b). To confirm penetration into the dermis of the animal (where the ISF is found), we dissected the skin tissue after MN insertion. Histological examination showed that the MN penetrated down to a depth of ∼700 μm from the skin surface, as shown in Figure S13c. Finally, the effect of the MN insertion on the skin tissue was evaluated by inserting the MN patch for 10 s, removing it, and taking pictures at the 5, 10, and 20 min after MN extraction (Figure S13d). Notably, we observed marks and skin recovery similar to those of the on-body tests in rats, with no signs of bruising, inflammation, or other alterations (Figure S13e).

The 12 pieces of rat skin were conditioned for 12 h at 4 °C (in the fridge) in solutions containing Lac concentrations of 1, 3, and 5 mM (4 skin pieces per concentration). Then, we used the MN patch for the transdermal detection of Lac. Prior to the MN-based measurements, a 3-point calibration graph of the patch was accomplished. The calibration graph was utilized to calculate the Lac concentration in each piece of skin from the recorded amperometric signals. After the MN-based measurements, the ISF inside each skin was collected by means of a custom-made system based on a hollow MN-hub, and the samples were analyzed by the Lactate Scout and IC. The results are collected in Table S3 (Supporting Information). Notably, the Lac content in the skin pieces conditioned with the same concentration was found to vary, as expected from the fact that Lac diffusion will differ in them owing to the different content in fat, thickness, and others.^38,39^

**Figure 4:**
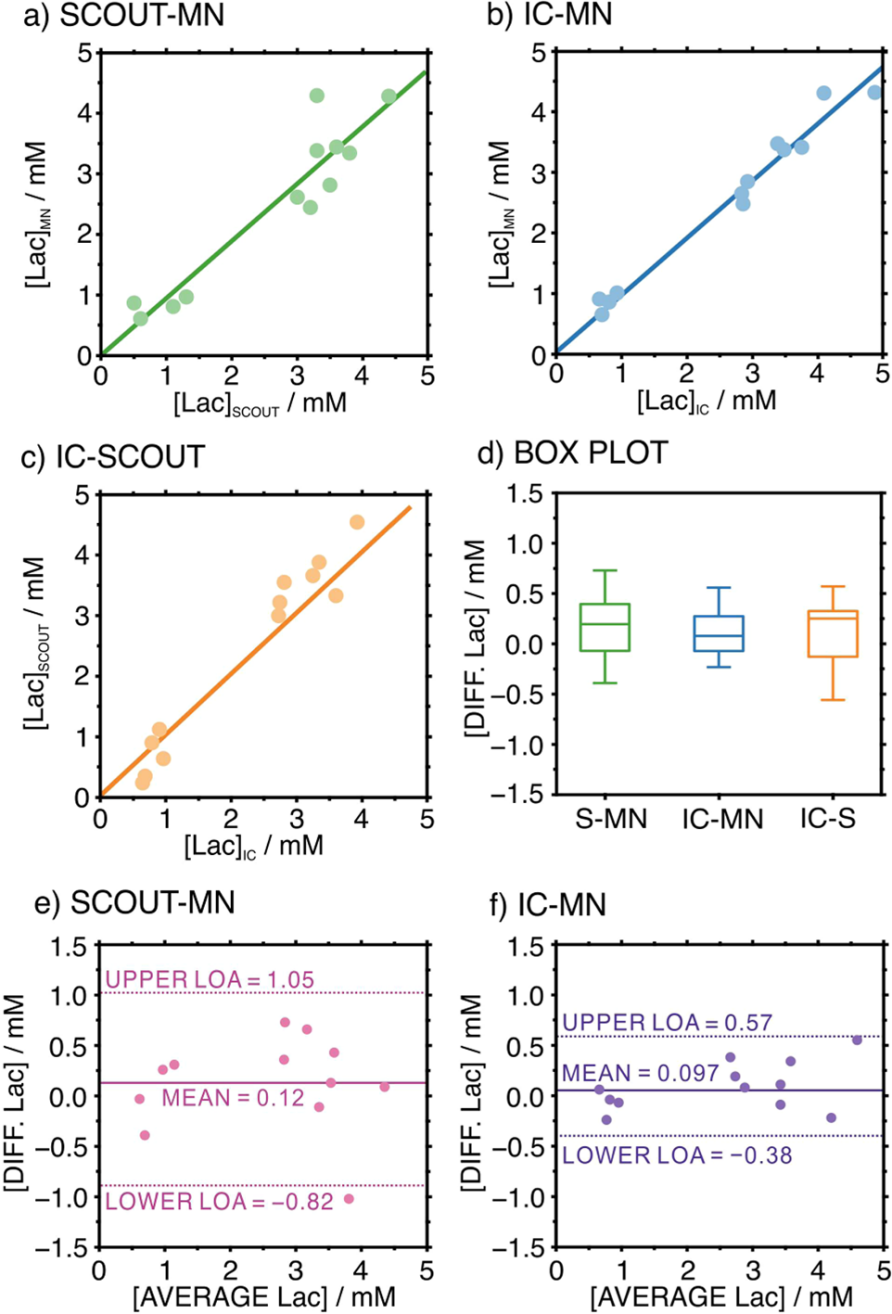
Correlation plots for Lac concentrations observed with (a) Scout and MNs, (b) IC and MNs, and (c) Scout and IC. (d) Paired sample *t*-test box plot. S = Scout. (e) Bland–Altman plot of the differences in the Lac values provided by the Scout and MNs. (f) Bland–Altman plot of the differences in the Lac values provided by the IC and MNs. IC: ion-chromatography.

Seemingly, the concentrations provided by the Lactate Scout were the ones that differed the most considering the three techniques. Nevertheless, when the correlations between the techniques were studied in pairs and calculating the Pearson correlation coefficient (Figure 4a–c), excellent agreements were observed in all the cases, and with statistically significant positive correlations. The intercepts were close to zero (0.04, 0.13, and 0.11 for MN-scout, MN-IC, and Scout-IC relationships, respectively), and the coefficients were higher than a threshold of 0.90 (0.94, 0.98, and 0.95 for MN-scout, MN-IC, and Scout-IC, respectively).

A two-tailed paired sample *t*-test was performed to determine whether the mean differences in Lac measurements between the utilized techniques analyzed in pairs were statistically significant or not. The calculated statistic *t* values (0.9, 1.4, and 1.0 for Scout MN, IC MN, and IC-Scout, respectively) were found to be lower than the critical value, *t*_critical_ = 2.2. Accordingly, no statistically significant differences were observed at the significance level of 95%. Moreover, the differences in Lac concentrations were analyzed by using a box and whisker plot (Figure 4d). The medians were close to zero (0.195, 0.08, and 0.25 mM for Scout MN, IC MN, and IC-Scout, respectively), with a small interquartile range (maximum = 0.43 mM). Figure 4e,f presents the distribution of Lac differences when measuring with the MNs and the Scout or IC. In both cases, a mean difference rather close to 0 mM was observed (0.12 mM for the Scout and 0.097 mM for IC). Then, the lower and upper limits of agreement at a 95% confidence level were relatively narrow, demonstrating the accuracy of the results provided with the MNs. Both the Scout and IC are suitable to validate MN-based Lac measurements. Notably, we selected the Scout for further measurements since it does not require sample storage, transportation, and pretreatment while needing a lower sample volume (0.5 μL).

**Figure 5:**
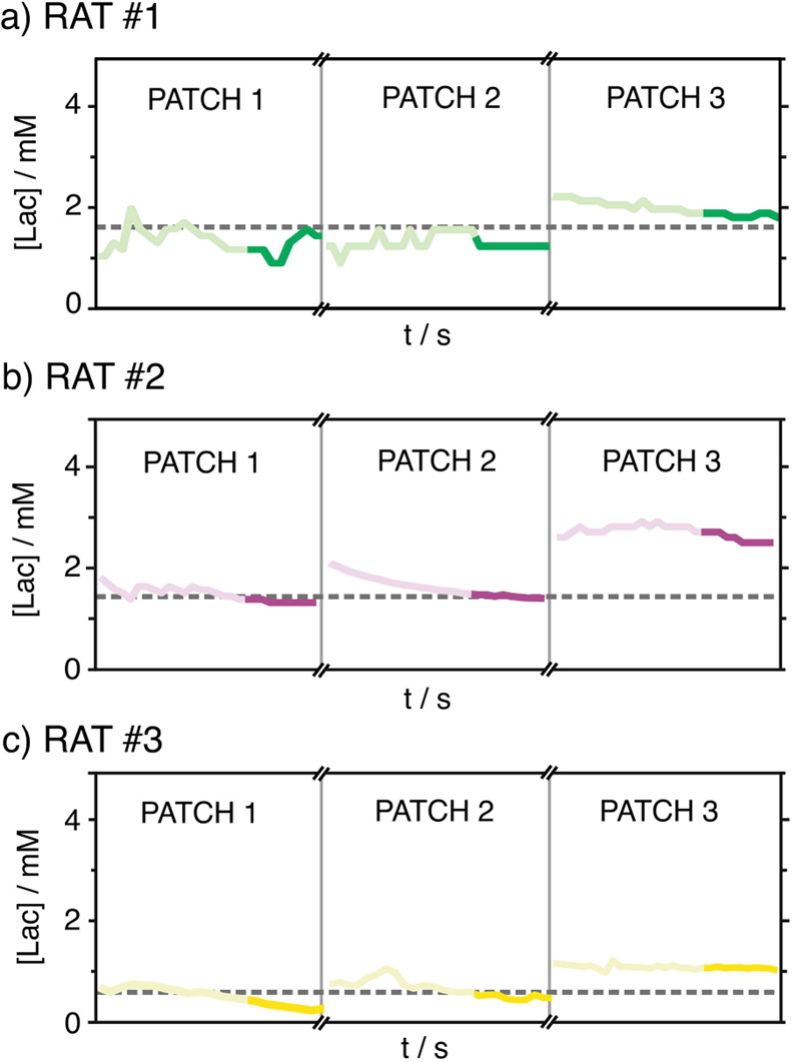
On-body Lac concentrations observed with three MN patches in euthanized rats #1 (a), #2 (b), and #3 (c), each measurement was accomplished for 30 s (total time scale). Subcutaneous Lac values are also included, represented by the dashed lines.

Next, we performed on-body measurements with the MN patch on three euthanized rats (rat #1–rat #3). After euthanasia, the rat’s back was shaved, and the MN patch was manually inserted. Three patches were used for each rat to obtain on-body Lac concentrations during 30 s (i.e., a total of 9 MN patches were used), converting the current signals by using a previous calibration graph in AISF. Due to different practical aspects, the total time needed for the MN-based measurements for each rat were ca. 70, 45, and 35 min. Figure 5 presents all the observed Lac profiles (3 patches per rat providing a measurement for 30 s each one), together with the discrete measurements performed in the subcutaneous ISF (dotted line). Despite our attempts to collect ISF samples after each on-body measurement, the obtained volume was not enough for the Lac analysis by means of either the Scout or IC. As an alternative, we implemented subcutaneous Lac measurements via an incision in the rat’s back with the Scout, directly contacting the fluid with the Lac sensing strip. This type of measurement has proven to be appropriate for the validation of other MN sensors.^40^ All of the results are summarized in Table S4 in the Supporting Information. Notably, average Lac concentrations were obtained from the last 10 s of each measurement (darker parts in Figure 5).

The Lac concentrations provided by the different MN patches used for the same rat were found to rather coincide between them, except for the Lac level shown by the third patch used in rat #2. Indeed, the average Lac concentration provided by that patch (2.7 mM, Table S4) was slightly outside the physiological range for blood Lac and, therefore, ISF Lac (from 0.3 to 2.5 mM).^5,41,42^ Accordingly, we considered such a measurement an outlier. Moreover, the Lac concentrations rather coincided with the subcutaneous Lac obtained by means of the Scout, with an average difference between both techniques of ca. 11%. Notably, for the third rat, the measurement of the subcutaneous ISF was not precise enough for a quantitative comparison with the values provided with the MNs. As the Lac level was below the LRR, the Scout provided a <0.5 mM value. Accordingly, these measurements were not used for the calculation of the averaged difference between both techniques, with the values provided by the MNs being between 0.3 and 0.9 mM (Table S4). Overall, subcutaneous Lac measurements with the Scout were well correlated with the ISF Lac provided by the MNs.

### In Vivo Measurements in Anesthetized Rats

**Figure 6:**
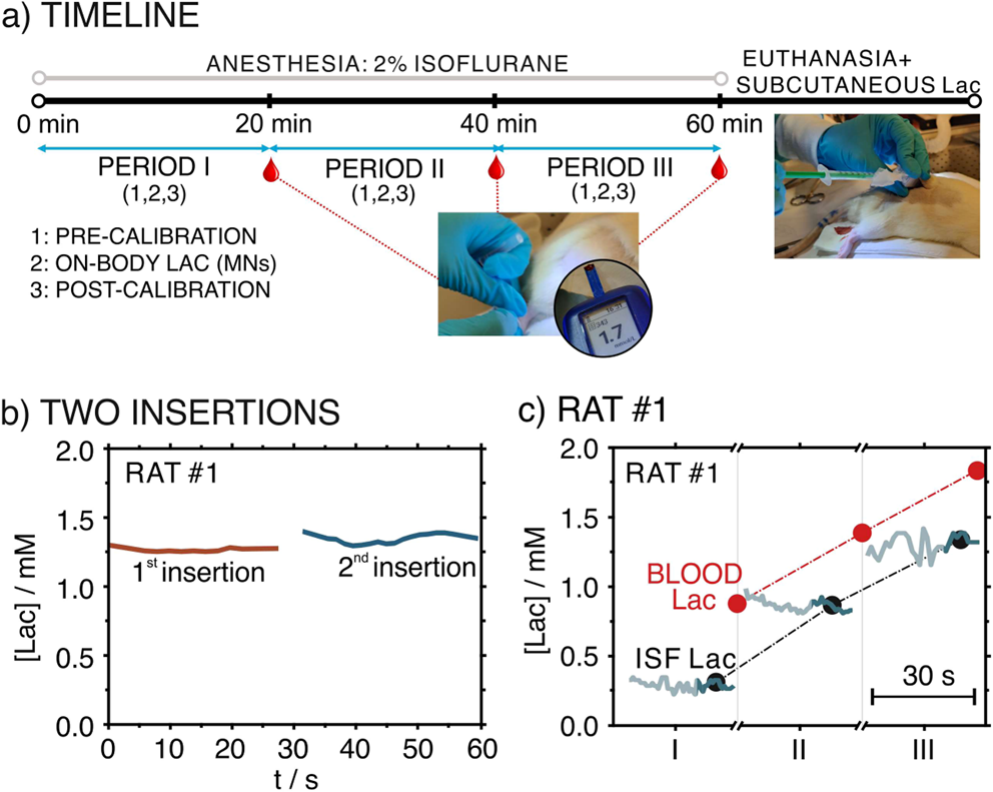
(a) Timeline of the experimental procedure followed for in vivo measurements in anesthetized rats. (1) precalibration; (2) on-body measurements; (3) post-calibration. (b) Dynamic Lac profile obtained with a Lac MN patch inserted twice in the back of rat #4. (c) Dynamic Lac concentrations recorded in periods I–III in rat #4. Black points indicate the Lac concentration averaged in the last 10 s of the recording (darker art of the concentration traces). Red points indicate blood lactate.

Before transitioning to clinical trials, we performed in vivo studies using rats. Validated in vivo measurements with the Lac patch were performed in five anesthetized rats (rat #4–rat #8). The experiments were approved by and conducted in accordance with the Uppsala Animal Ethics Committee (5.8.18-18873/2018). Figure 6a shows the timeline of the measurements. During 1 h, the on-body Lac measurements were performed: from 0 to 20 min, from 20 to 40 min, and from 40 to 60 min (periods I–III) using different MN patches. Notably, we evaluated equivalent determinations with two patches (rat #4 in period I), the same patch pierced in two close back positions (rat #4 in period III) as well as “medium-term” measurements (5 min, rat #6 in period I). After each period, i.e., at ca. 20, 40, and 60 min, blood was collected from the rat’s vena saphena, which was immediately analyzed with the lactate Scout. After that, the rat was euthanized and subcutaneous ISF was collected and analyzed with the Scout. The results are given in Table 1.

**Table 1:** Lactate Measurements in Anesthetized Rats

rat #	period	Lac (mM)		
		MN patch	blood	subcutaneous
4	I^a^	0.32	0.9	<0.5
		0.33		
	II	0.79	1.4	
	III^b^	1.34	1.8	
		1.37		
5	I	0.54	0.9	<0.5
	II	0.55	1.2	
	III	0.97	1.7	
6	I^c^	0.72 ± 0.05	0.7	<0.5
	II	d	<0.5^e^	
	III	d	1.0	
7	I	0.72	1.5	<0.5
	II	1.48	1.4	
8	I	0.65	1.7^e^	<0.5
	II	0.88	0.7	
	III	0.93	1.9^e^	

a Measurements with two patches at the same time.

b Measurements with two insertions of the same patch.

c Medium-term measurements (5 min).

d Response out of the LRR.

e Not enough blood was collected for the measurements.

The response of the sensor was recorded until the steady-state potential was reached, and lactate concentrations were calculated as the average of the last 30 s approximately. The total time of each insertion did not exceed 2 min, except for rat #6, in which the signal was recorded for 5 min.

Importantly, neither cytotoxicity risk nor response deterioration (drift) caused by component leaching from the sensing element in the MNs (both the WE and RE) is expected during the time frame used in the herein tests. Regarding biocompatibility, previous cytotoxicity studies performed in our research group revealed that MNs covered with polymers of similar compositions do not cause cell damage from 0 to 96 h when incubated at different conditions with fibroblasts.^43^

Analyzing first the results observed in rat #3, consecutive measurements using two patches (period I) revealed very similar Lac concentration, with a variation of 3%. Similarly, the variation found for measurements performed with the same patch through two subsequent insertions (period III) rather agreed in the Lac profile (Figure 6b) and the average concentration, with a variation of 2%. Interestingly, both ISF and blood Lac were found to increase with increasing analysis time, with always blood Lac being higher than the ISF one (Figure 6c, black and red points). A similar trend was found in rat #5 and rat #6, being less evident in rat #7 and rat #8. Moreover, in rat #5 (period I) as well as in rat #6 (period I), the utilization of the Lac MN patch to perform continuous measurements during 5 min was studied, revealing a rather stable signal with a standard deviation of 0.05 mM, the ca. 5% of the averaged Lac concentration.

**Figure 7:**
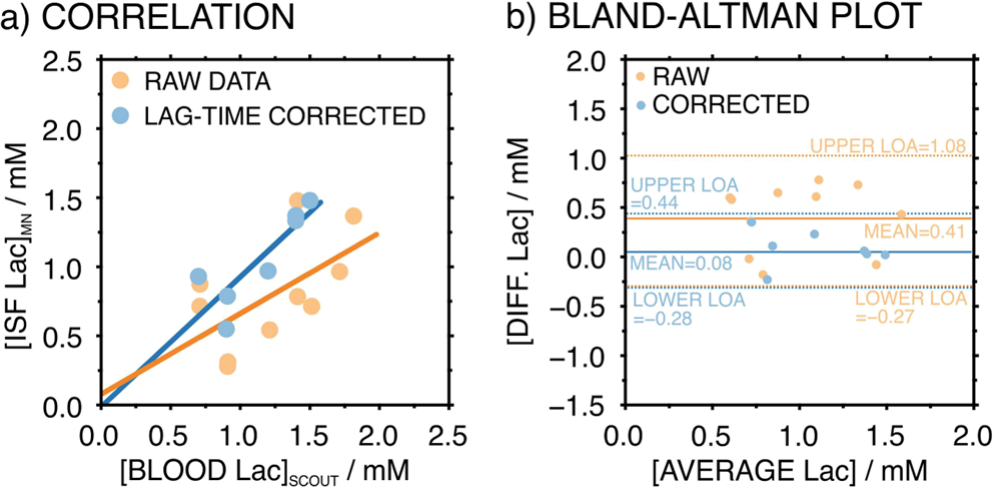
(a) Correlation graph of ISF Lac monitored by the MN sensor patch and blood Lac measured by the Lac Scout. (b) Bland–Altman plot of the difference between MN sensor patch measurements and blood Lac levels. Color code: orange, raw data; blue, lag-time corrected.

While the increase in blood Lac is likely explained by an effect of isoflurane anasthesia,^44^ a closer inspection of ISF and blood Lac values informed that blood Lac better coincided with ISF Lac in the next period than in the same one. In other words, blood Lac in period I was close to ISF Lac in period II, and blood Lac in period II was close to ISF Lac in period III. Figure 7a depicts the correlations between ISF and blood Lac without and with consideration of the described lag situation (the raw data are provided in Table S5, Supporting Information). Pearson coefficients of 0.61 and 0.85 were observed, respectively, confirming that Lac concentrations in both fluids are highly correlated when the lag time is considered (a cutoff value of 0.80 to ensure the correlation). Moreover, the intercepts of the lines were found to be 0.088 and 0.015 mM, confirming that the Lac relationship between two fluids is even more consistent when the lag time is considered (i.e., intercept almost equal to zero).

Our findings support the hypothesis that there is a lag time to visualize changes in Lac concentration in ISF with respect to those happening in blood. Indeed, we quantified such a time to be ca. 10 min, attending to the exact experimental timeline registered for each of the five rats. A literature search revealed that some reports claimed a short (and thus no significant) lag time for Lac diffusion from blood to ISF (<5 min).^21^ However, other studies concluded a varying lag time from 5 to 10 min^45,46^ This lag time has been observed for other small molecules, such as glucose, in rats as well as in humans.^47^ Notably, discrepancies may arise between individuals and special circumstances, such as medication, exercise, and diet. In our case, the decrease in heart rate and blood pressure caused by isoflurane anesthesia may contribute to slowing down the process of Lac diffusion from the bloodstream to the ISF via venous capillaries.^48^

Despite trying subcutaneous Lac measurements after euthanasia, the values provided by the Scout were always below its LOD, and thus, they could not be used for a proper quantification. Furthermore, the extremely low volume of ISF that we obtained did not allow analysis by IC. As a result, we did consider blood measurements to validate ISF concentrations observed with the MN patch. Thus, a Bland–Altman analysis (Figure 7b) was used for the statistical analysis of the data. A relatively low mean difference of 0.08 mM was obtained with the proposed lag-time correction, being much higher and more significant when no correction is accomplished (0.41 mM). The limits of agreements (LoAs) were calculated to be −0.28 and 0.44 mM as well as −0.27 and 1.08 mM with and without lag time correction. The LoAs for the case considering the lag time correction are within the clinically accepted accuracy standards considering that Lac ISF levels are expected to be in the range of 0.3 to 2.5 mM (i.e., ± 20%).^4^

## Conclusions

We have demonstrated a MN-based sensor for accurate detection of Lac in the ISF in the 0.25–35 mM range. Thus, not only the physiological range is covered (0.3–2.5 mM) but also concentrations related to some specific diseases as well as physically active conditions. The stability, robustness, and other analytical performances were evaluated by a series of in vitro and ex vivo experiments. The MN sensor was integrated into a miniaturized, lightweight, and portable device that could be easily implemented for on-body measurements. The data acquisition process is fully automated, with the data being collected and then wirelessly transmitted to the smartphone application. We were able to obtain real-time Lac measurements in eight rats. The MNs’ readings were in strong agreement with those obtained using reference methods to detect ISF and blood Lac. This study provides two significant contributions to the MN sensing technology field. First, the feasibility of using MN for lactate in vivo measurements in ISF is demonstrated. Second, the first comprehensive validation of Lac ISF measurements as well as the ISF-blood correlation were realized.

Regarding the latter, importantly, a lag time of 10 min was observed. The outcomes from in vivo measurements in anesthetized rats revealed the effect of increasing Lac concentrations in both blood and ISF under the anesthesia effect. Overall, the developed Lac MN patch has displayed great potential for a variety of clinical and physiological applications, as well as more basic life science research.

## Supporting Information Available

Experimental details; state of the art of Lac MNs; calibration parameters at different conditions; results in the ex vivo experiments; lag time corrected data in the in vivo experiments; analytical figures of merit; and additional real images (PDF)
