# Supplementary material for: Intradermal Lactate Monitoring Based on a Microneedle Sensor Patch for Enhanced In Vivo Accuracy
Source: ACS Sens. 2024 May 23;9(6):3115–25. doi: 10.1021/acssensors.4c00337 (PMC11217941; doi:10.1021/acssensors.4c00337)
Supplement: Supplementary file 1 — se4c00337_si_001.pdf [file se4c00337_si_001.pdf]

Supporting information for:

Intradermal Lactate Monitoring Based on a Microneedle Sensor Patch for Enhanced In Vivo Accuracy

Qianyu Wang[1], Águeda Molinero-Fernández[1,2], Qikun Wei[1], Xing Xuan[1,2], Åsa Konradsson-Geuken[3], María Cuartero[1,2,*] and Gastón A. Crespo[1,2,*]

[1]*Department of Chemistry, KTH Royal Institute of Technology, Teknikringen 30, SE-114 28, Stockholm, Sweden.*
[2]*UCAM-SENS, Universidad Católica San Antonio de Murcia, UCAM HiTech, Avda. Andres Hernandez Ros 1, 30107, Murcia, Spain.*
[3]*Section of Neuropharmacology and Addiction Research, Department of Pharmaceutical Biosciences, Uppsala University, SE-751 05, Uppsala, Sweden.*

(*) Corresponding Authors: Maria Cuartero (mariacb@kth.se). Gaston Crespo (gacp@kth.se)

Table of Contents

1. Experimental Section......3
1.1. Reagents, materials, and instrumentation......3
1.2. Fabrication of the MN patch......3
1.3. Fully integrated wearable system for intradermal lactate monitoring......3
1.4. *Testing of penetration force*......4
1.5. *In vitro* analyses......4
1.6. *Rat-based* experiments......4
1.7. IC measurement......5
2. Tables......6
Table S1......6
Table S2......7
Table S3......7
Table S4......8
Table S5......8
3. Figures......9
Figure S1......9
Figure S2......9
Figure S3......10
Figure S4......10
Figure S5......11
Figure S6......11
Figure S7......11
Figure S8......12
Figure S9......12
Figure S10......12
Figure S11......13
Figure S12......13
Figure S13......13
4. References......14

1. Experimental Section

1.1. Reagents, materials, and instrumentation. Lactate oxidase was purchased from Sorachim SA, Switzerland (LOx, EC 1.13.12.4, 111 $U \cdot mg^{-1}$). Sodium chloride (CAS-7647-14-5), potassium chloride (CAS-7447-40-7), sodium phosphate dibasic (CAS-10028-24-7), potassium phosphate monobasic (CAS-7778-77-0), iron (III) chloride (CAS-7705-08-0, >97% purity), potassium ferricyanide(III) (CAS-13746-66-2, >98% purity), glacial acetic acid (CAS-64-19-7), low molecular weight chitosan (CAS-9012-76-4), high molecular weight poly(vinyl chloride) (PVC, CAS-9002-86-2), bis(2-ethylhexyl) sebacate (DOS, CAS-122-62-3, ≥97% purity), tetradodecylammonium tetrakis(4-chlorophenyl)borate (ETH 500, CAS-100581-42-8), bovine serum albumin (BSA, CAS-9048-46-8), calcium chloride (CAS-22691-02-7), sodium bicarbonate (CAS-144-55-8), magnesium chloride (CAS-7786-30-3), polyurethane (PU, CAS-51-79-6), polyvinyl butyral (PVB, CAS-63148-65-2), tetrahydrofuran (THF, CAS109-99-9), L-ascorbic acid (CAS-50-81-7), and hydrogen peroxide (CAS-7722-84-1) were purchased from Sigma-Aldrich. Hydrochloric acid (CAS-7647-01-0), sodium pyruvate (CAS-113-24-6), uric acid (CAS-69-93-2), D-(+)-glucose anhydrous (CAS-50-99-7), sodium L-lactate (CAS-867-56-1), and urea (CAS-57-13-6) were purchased from Alfa Aesar. Sodium hydroxide (CAS-1310-73-2), agarose DNA grade, and ethanol absolute (CAS-64-17-5) were purchased from VWR. Silver/silver chloride (Ag/AgCl) and carbon sensor paste were obtained from Sun Chemical, UK distributor. All solutions were prepared using double-deionized water of 18.2 $M \cdot cm^{-1}$ from the Milli-Q water systems, Merck Millipore. Sodium phosphate buffer (0.1M, pH 7.4) was created by combining NaH_2PO_4 and Na_2HPO_4 solutions. Phosphate buffered saline solution (PBS, 0.01M, pH 7.4) was prepared from the mixed dissolution of NaCl, KCl, Na_2HPO_4, and KH_2PO_4. Artificial interstitial fluid (AISF, pH 7.4) was prepared as previously described.[1,2] Briefly, the composition of AISF was 20 $g \cdot L^{-1}$ BSA, 3.5 mM KCl, 1.5 mM $CaCl_2 \cdot 2H_2O$, 0.7 mM $MgCl_2$, 140 mM NaCl, 26 mM $NaHCO_3$, 1.7 mM NaH_2PO_4.

1.2. Fabrication of the MN patch. The material of the substrate for fixation of microneedles being used was silicone rubber (Ecoflex 00-50 platinum cure, USA), which is a commercially procurable material widely employed in the production of prosthetic appliances and cushioning for orthotic uses. The substrate was fabricated by mixing equal volumes of solutions labelled as ‘Part A’ and ‘Part B’ of the commercial pourable silicon rubber and filling a 3D printed mold of 13 mm of diameter and 1 mm of depth with the resulting mixture. Afterwards, the substrate was allowed to cure at room temperature for 3 h, according to the manufacturer instructions. The mold was designed with AutoCAD. The adopted stainless steel microneedle (SST-MN) (Dermaroller, local supplier, Sweden) originates from medical-grade microneedle dermal rollers that were off-the-shelf products initially devised for medical aesthetics applications.

Lac MN sensor patch is composed of a silicon rubber substrate (Ecoflex 00-50 platinum cure, USA) with two modified MNs: a working electrode (MN WE) and a pseudo counter/reference electrode (MN CE/RE). The commercial stainless steel MNs (1000 µm in length, 200 µm diameter in the body and 25 µm in the tip) were dip-coated with the corresponding ink (Carbon for the MN WE and Ag/AgCl for the MN CE/RE) and then cured in the oven (120 °C, 10 min). The MNs were fixed in the flexible substrate using Loctite Super Glue (Henkel Norden AB).

1.3. Fully integrated wearable system for intradermal lactate monitoring. Summarily, the nRF52832 microcontroller unit (MCU) serves as the central processing unit, controlling the overall system operation. The MCU is responsible for executing program logic, performing calculations, and communicating with various peripherals and external devices. The LMP91000 integrated circuit is a specialized chip designed for electrochemical (amperometry) sensing applications. The integrated circuit provides signal conditioning and amplification for accurate measurement and includes programmable features to optimize sensor performance (control the applied voltage and range of signal recording). The MCU sends commands and configuration data to the LMP91000 integrated circuit and receives the sensor data for further analysis and allows wireless transmission through Bluetooth. A voltage regulator is used to

regulate the power supply to the components in the system. It ensures a stable and consistent voltage level for proper operation. A Li-ion battery is included to provide a portable and rechargeable power source for the system.

1.4. *Testing of penetration force.* A texture analyzer (CT3, VWR) was used to analyze the penetration force applied to the MN sensor patch during insertion into hydrogels or rat skins. Skin-mimicking hydrogels were prepared from the recipe that was previously reported elsewhere.[3] The schematics of the experimental setup for penetration force testing was shown in **Figure S5a**. A trigger force (i.e., until detecting this force, the device starts to record the force following a set penetration depth) was set to 0.049 N and 0.98 N for hydrogels and rat skins, respectively. The penetration depth was set to 0.7 mm that is slightly larger than the length of the MNs (~0.6 mm). The microneedle sensor patch was placed on top of the hydrogels on a fixture base table (TA-BT-KIT). Then, the probe of the texture analyzer was slowly moving downwards, until detecting a trigger force. Afterwards, the probe pushed the MN sensor to insert into the hydrogel for 0.7 mm and penetration forces were recorded. The peak force will be available on the device display. Five MN sensor patches were used for the testing.

1.5. *In vitro* analyses. A stationary Autolab potentiostat (PGSTAT302N, Metrohm) , in conjunction with an Ag/AgCl/saturated KCl reference electrode (RE, model 6.0733.100, Metrohm), a platinum counter electrode (CE) (CE, model 6.1248.000, Metrohm), and a specially tailored microneedle working electrode (MN WE), was employed. The production of the MN RE followed a meticulously investigated methodology previously employed by our research group.[1,2,4] Two chronoamperometric techniques were employed to achieve specific measurements of lactate, employing an applied potential of −0.1 V relative to an Ag/AgCl reference electrode. For fixed-time chronoamperometry, the sensor underwent a two-minute incubation in the solution prior to the application of a constant negative potential and afterward, the current-time curve was recorded for 30 seconds. In the method of continuous amperometry, the solution experiences uninterrupted stirring, while lactate is incrementally introduced into the solution, the corresponding step curve illustrates the current arising attributed to alterations in lactate concentrations.

A dual extruder 3D printer was employed to fabricate the external device encasing, functional gadgets, pouring molds, and storage containers for the sensor patch (Ultimaker 3 B.V., Netherlands). An inverted optical microscopy setup (Nikon Eclipse Ti2, Japan) was utilized to photograph the detailed imagery of the microneedles' morphology and structure. NX50 Cryostat (Epredia, USA) was employed for performing the histological cuts. Validation of blood lactate was accomplished using a lactate scout (Lac Scout) readily available in the market (Lactate Scout 4, EKF diagnostics, UK). Additionally, an immediate on-site analysis of a small amount of ISF using the Lac Scout was completed concurrently for comparison with values obtained from the Lac MN sensor. In the laboratory context, the utilization of ion chromatography (IC) (850 Professional IC, Metrohm AB, Sweden) as a second gold standard method served to establish a definitive reference point.

1.6. *Rat-based* experiments. For experiments in rats, a home-made, portable potentiostat with built-in wireless data transmission functionality was conveniently utilized to acquire instantaneous, dynamic current measurements through our custom smartphone application. Important information including rat age, weight, gender, species/breed, health status, living conditions, and treatments were registered.

Ex vivo intradermal experiments on euthanized rats were performed at the Experimental Research and Imaging Centre (KERIC, Karolinska Institute, Sweden) with the assistance of the surgical directors and laboratory technician, by adopting rats that were initially subjected to euthanasia for other research objectives and were not deliberately chosen for our particular experiment. Male DA DsRed-Express-2 rats donated from KERIC were seven months old.

The *In vivo* experiments were carried out in rats under anesthesia at Uppsala University (UU) with approval from the Uppsala Committee on Ethics of Animal (Dnr 5.8.18-18873/2018, DOUU-2020-025). Adult male Wistar rats (Envigo, Horst, The Netherlands), weighing approximately 180 grams on arrival (~6 weeks of age). All animals were maintained in groups of 4 under standard laboratory conditions (room temp of ~21 Celsius degrees and relative humidity of 55-65 %) with a 12 h reversed light / dark cycle (lights on at 7 PM) and full access to food and water. In the beginning of the experiment, a 2% concentration of isoflurane flow was used to anesthetize the rats during the on-body measurements. A heating pad was prepared and positioned beneath the rats to sustain their core body temperature. Eye drops were administered to the rats to protect their eyes from desiccation. In the event of a reduction in body temperature detected on the dorsal surface of the rat, supplementary thermal source was administered. An area of fur of 5 x 5 cm was shaved from the back, sanitized using alcohol, and subsequently inserted with the Lac MN connected apparatus. The vital signs of the anesthetized rats were closely monitored. Two significant indexes, namely pedal reflex and respiratory rate were examined prior to the tests to determine if the rats were showing signs of waking up from anesthesia. If not, the isoflurane flow was increased and the rats were allowed to wait for a longer period before the experiments were conducted. After the MN-based experiment, each animal got a lethal dose of isoflurane followed by decapitation Skin from euthanized rats was collected and stored frozen in 0.1 M PBS solution for subsequent studies.

1.7. IC measurement. For lactate determination, carbonate buffer (1.0 mM $NaHCO_3$/ 3.2 mM Na_2CO_3), and 100 mM H_2SO_4 were employed as eluent and suppressor solutions. A flow rate of 0.8 mL/min was selected. For calibration in IC, 1 μM, 2 μM, 3 μM, 4 μM, and 5 μM Lac standard solutions were prepared in milli-Q water. ISF samples were diluted in milli-Q water (1:1000) prior to IC tests.

2. Tables

Table S1. Characteristics of the MN-based sensors for lactate detection reported in the literature. Abbreviations. Rh=Rhodium; C=Carbon; LOx=Lactate Oxidase; PEI = polyethyleneimine; MWCNTs = Multi-wall Carbon Nanotubes; MB = Methylene Blue; GP = Graphite Powder; MO = Mineral Oil; PB = Prussian Blue; GA = Glutaraldehyde; Chi = Chitosan; PVC = Polyvinyl Chloride; BSA = Bovine Serum Albumin; SiNW-FET = Silicon-Nanowire Field-Effect Transistors; Pt = Platinum; AuNPs = Gold Nanoparticles; PDA-NSs = Polydopamine Nanospheres; PANI = Polyaniline; polyHEMA = Poly(2-hydroxyethyl methacrylate); PEG = Polyethylene Glycol; PDMS = Poly(dimethylsiloxane); PVA = Polyvinyl Alcohol; ETH 500 = Tetradodecylammonium tetrakis(4-chlorophenyl)borate.

#REF	Electrode Modification	LRR	LOD	Detection Method	Applied Potential (V) Versus Ag/AgCl	Microneedle Type	Microneedle Materials	Validation	Ex vivo	In vivo
9	Rh/C + LOx/PEI	0–8 mM	0.42 mM	Amperometry	−0.15	Hollow	Eshell 200 acrylate-based polymer	–	–	–
21	Rh/C + LOx	–	–	Amperometry	−0.15	Hollow	E-shell 300 acrylate-based polymer	–	–	–
10	Rh/C + LOx/PEI + Lipdure	0–8 mM	0.42 mM	Amperometry	−0.15	Hollow	E-shell 300 acrylate-based polymer	–	–	–
11	MWCNTs + MB + LOx	10–200 μM	2.4 μM	Amperometry	+0.195	Solid	Gold-coated polycarbonate	–	–	–
12	MWCNTs + MB + LOx	10–100 μM	3 μM	Amperometry	+0.195	Solid	Gold-coated polycarbonate	–	–	–
13	GP/MO + PB + LOx/GA + Chi + PVC/surfactant	0–10 mM	–	Amperometry	−0.1	Hollow	Carbon paste packed electrode	–	–	–
41	BSA+GA+LOx	1–30 (PBS); 5–30 (Blood) mM	–	DPV	−0.1 to 0.8	Solid	Gold nanoparticles-coated vinyl	–	✓ (Blood/Plasma)	–
15	SiNW-FET	0–1 mM	–	Amperometry	−0.3	Solid	Silicon-on-insulator	–	–	✓ (Rat)
14	Pt + AuNPs/PDA-NSs	0.375 uM–12 mM	50 μM	DPV	−0.2 to 0.8	Solid	Platinum	–	–	✓ (Cell)
17	Au + PANI + Lox + Nafion + poly HEMA	0–200 mg/dL	–	CV	−0.4 to 0.6/0.35	Solid	Polycarbonate	✓ (Blood)	–	✓ (Human)
18	LOx + PVC/surfactant	0–15 mM	–	Amperometry	0.6	Solid	PMMA	✓ (Blood)	–	✓ (Human)
19	Ptblack+LOx/glycerol/PEG/BSA+ Nafion	–	–	Amperometry	0.2	Solid	Polycarbonate	✓ (Blood)	–	✓ (Human)
20	PDMS/CNT/CNC + molecularly imprinted	25 – 150 mM	0.07 mM	CV	–0.5–0	Solid	PDMS	–	–	–
[5]	Non-enzymatic	0–10 mM	0.29 mM	Amperometry	0.45	Solid	Ti/Cu	–	–	–
[6]	PDMS/PVA	0–12 mM	–	Colorimetry	–	Solid	PDMS	–	–	✓ (Rat)
This paper	LOx + PVC/ETH 500	0–35 mM	0.249 mM	Amperometry	–0.1	Solid	SST	✓ (ISF)	✓ (ISF/Blood)	✓ (Rat)

Table S2. Analytical parameters of the MN-based Lac sensor obtained when using different configurations for the reference and counter electrodes.

	Slope (nA mM^{-1})	Intercept (nA)	R^2
Configuration 1	−40.69	−27.78	0.994
Configuration 2	−43.13	−18.78	0.996
Configuration 3	−39.48	−22.09	0.994
Configuration 4	−45.74	−47.18	0.992

Configuration 1: commercial RE and CE
Configuration 2: MN RE and commercial CE
Configuration 3: pseudo commercial CE/RE
Configuration 4: pseudo MN CE/RE

Table S3. Lac measurements in pieces of rat skin. Values are provided in mM.

Preconditioning Lac solution: 1 mM				
**Method**	**Skin #1**	**Skin #2**	**Skin #3**	**Skin #4**
MN Sensor	1.0	0.9	0.6	0.8
Lac Scout	1.3	0.5	0.6	1.1
IC	0.9	0.7	0.7	0.8

Preconditioning Lac solution: 3 mM				
**Method**	**Skin #1**	**Skin #2**	**Skin #3**	**Skin #4**
MN Sensor	2.8	2.6	3.4	2.5
Lac Scout	3.5	3.0	3.8	3.2
IC	2.9	2.8	3.5	2.9

Preconditioning Lac solution: 5 mM				
**Method**	**Skin #1**	**Skin #2**	**Skin #3**	**Skin #4**
MN Sensor	4.3	3.4	3.5	4.3
Lac Scout	3.3	3.3	3.6	4.4
IC	4.9	3.8	3.4	4.1

Table S4. On-body Lac measurements in euthanized rats.

Rat #1			
**MN Patch**		**Lac Scout**	**Diff. MN-Scout (%)**
**Patch number**	**Lac (mM)**	**Subcutaneous Lac (mM)**	
1	1.4	1.6	13
2	1.3		19
3	1.8		13

Rat #2			
**MN Patch**		**Lac Scout**	**Diff. MN-Scout (%)**
**Patch number**	**Lac (mM)**	**Subcutaneous Lac (mM)**	
4	1.2	1.3	8
5	1.4		8
6[a]	2.7		–

Rat #3			
**MN Patch**		**Lac Scout**	**Diff. MN-Scout (%)**
**Patch number**	**Lac (mM)**	**Subcutaneous Lac (mM)**	
7	0.3	<0.5[b]	–
8	0.4		–
9	0.9		–

[a]Outlier. [b]A value lower than the measurement range indicated for the Lactate Scout.

Table S5. Paired points used for correlation plot with lag-time correction

Lag-time corrected (n = 7)		
**Blood Lac from Scout (mM)**	**ISF Lac from MN (mM)**	**Rat number**
0.9	0.79	Rat #1
1.4	1.34	Rat #1
1.4	1.37	Rat #1
0.9	0.55	Rat #2
1.2	0.97	Rat #2
1.5	1.48	Rat #4
0.7	0.93	Rat #5

3. Figures

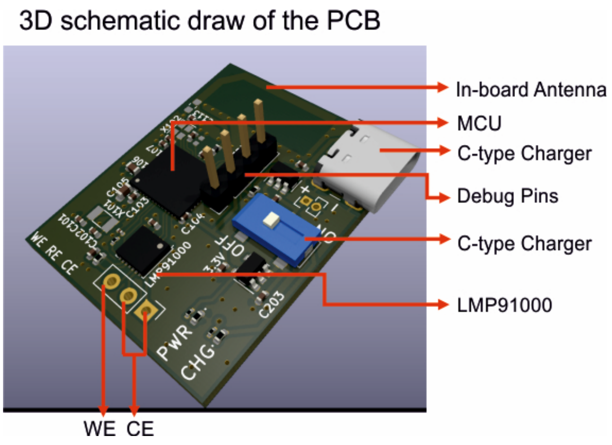

Figure S1. The schematic diagram of the PCB.

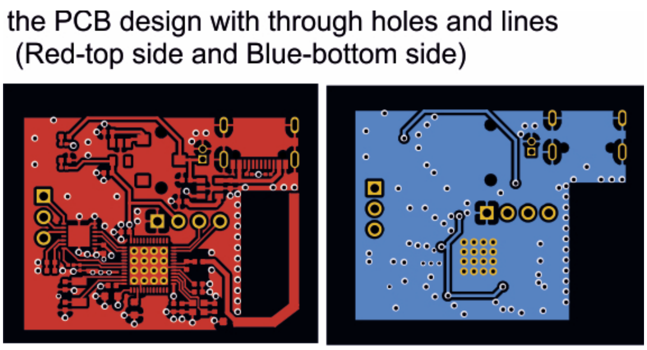

Figure S2. The printed wire layout of the PCB.

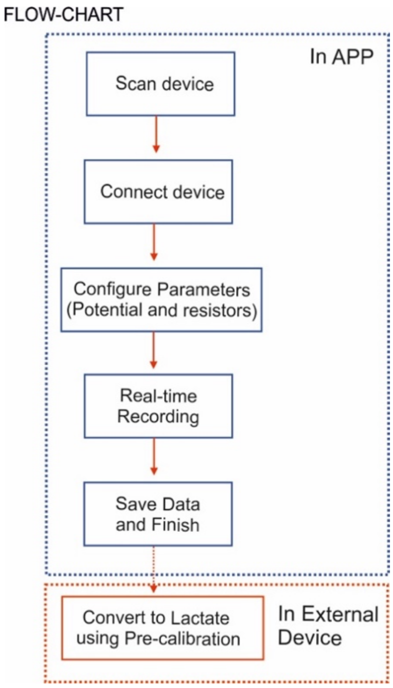

Figure S3. The workflow chart of the phone application.

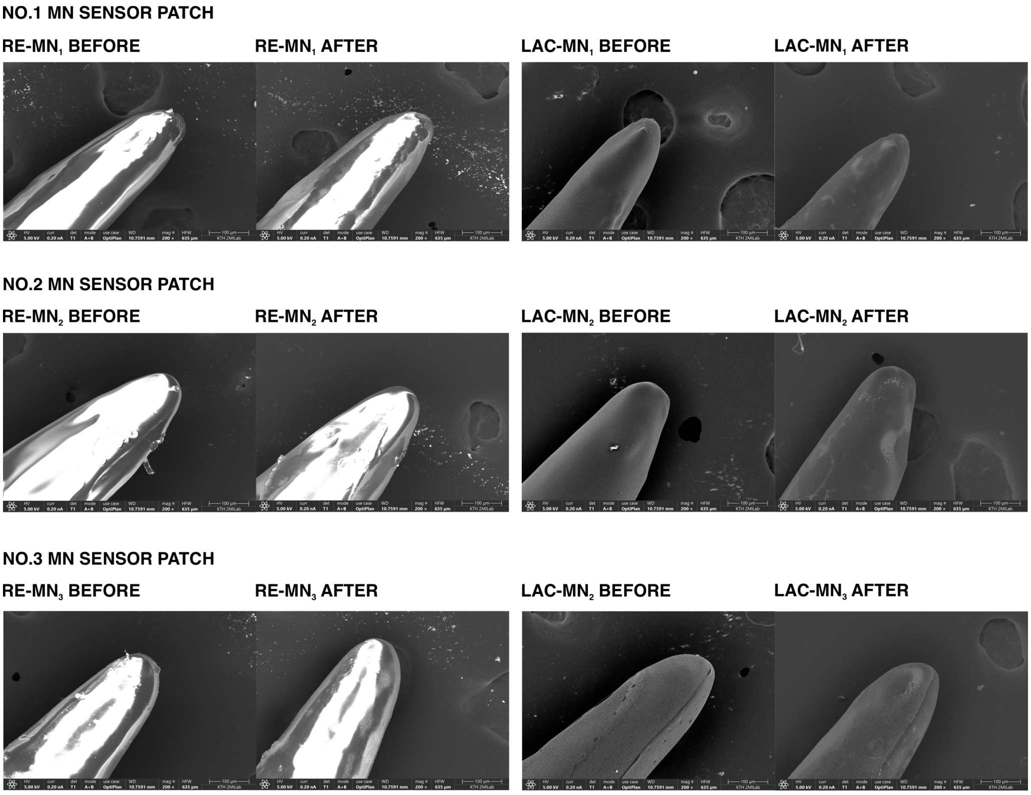

Figure S4. SEM images of the RE-MN and Lac-MN before and after insertions into the rat skin.

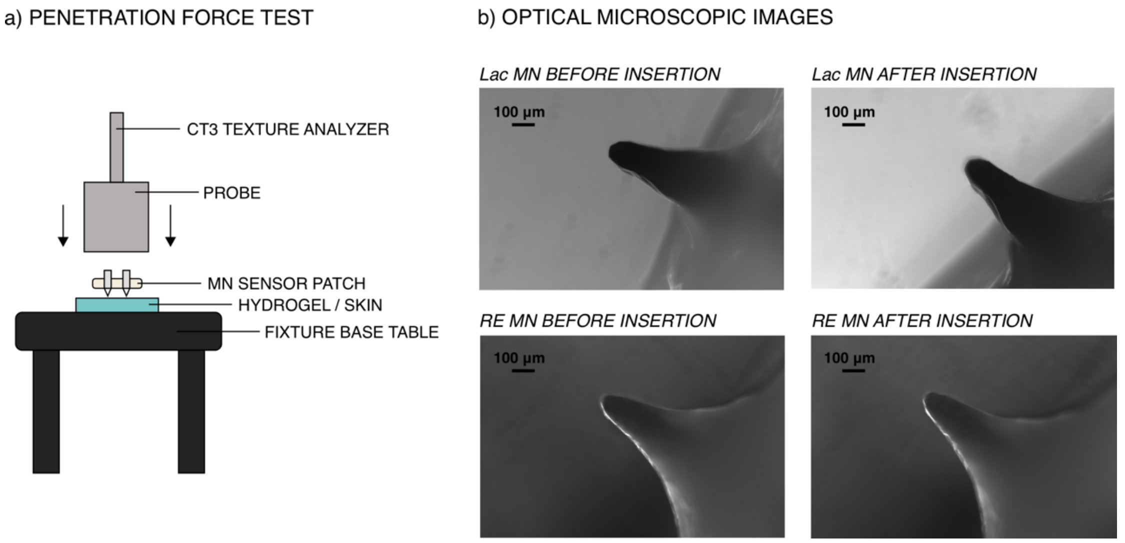

Figure S5. (a) Experimental setup for testing the required penetration force of the MN sensor patch into hydrogels / rat skins using a texture analyzer. (b) Optical microscopic images of the MNs before and after penetrations into the hydrogels and rat skins.

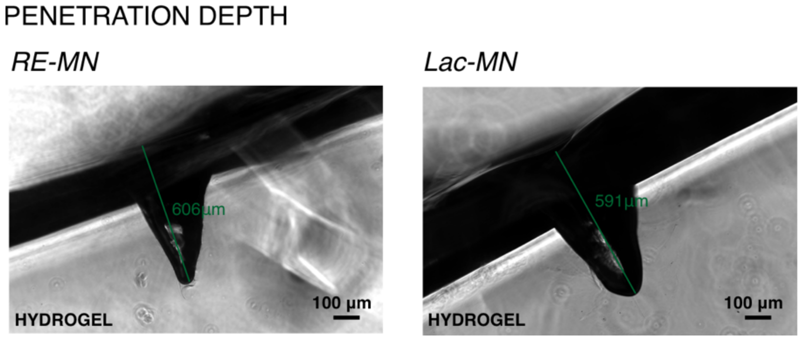

Figure S6. Penetration depth of the MN sensor patch during insertion into hydrogels.

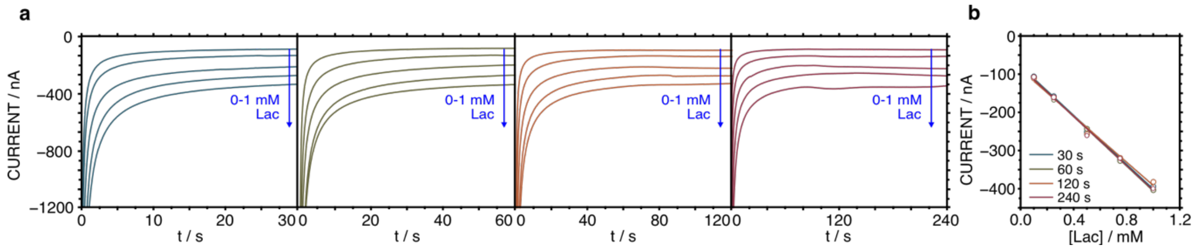

Figure S7. (**a**) Chronoamperometric responses of the MN patch recorded at 30s, 60s, 120s, and 240s, when measuring separate Lac solutions of increasing concentrations. (**b**) The corresponding calibration graph. The standard deviations in the slope and intercept were 2.7% and 3.5%.

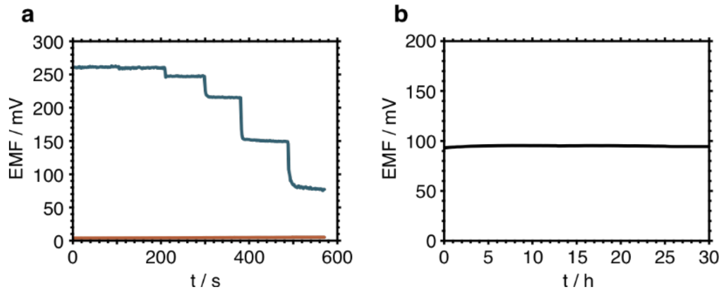

Figure S8. (**a**) Dynamic potentiometric responses of the RE-MN (orange) and the Ag/AgCl-MN (blue) towards increasing concentrations (10^{-5}, 10^{-4}, 10^{-3}, 10^{-2}, 10^{-1} M) of KCl. (**b**) Stability of the RE-MN in AISF for 30 hours.

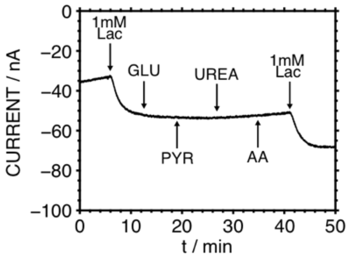

Figure S9. Dynamic amperometric response of the MN patch facing (in this order) 1 mM Lac, 6 mM glucose (GLU), 120 µM pyruvate (PYR), 7 mM urea (UREA), 100 µM ascorbic acid (AA), and 2 mM Lac.

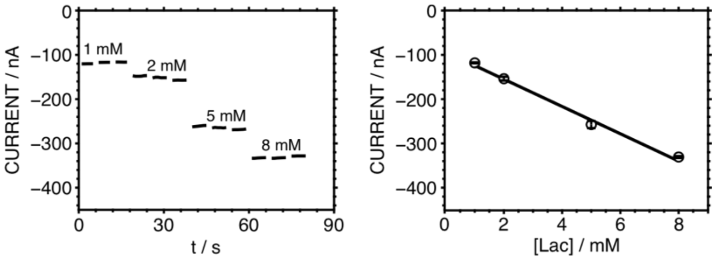

Figure S10. *Left:* Repeatability of the response of the MN patch by conducting consecutive measurements of solutions containing 1 mM, 2 mM, 5 mM and 8 mM of Lac solution. The last five seconds of the response were plotted in the figure. *Right:* the corresponding calibration graph (error bars corresponding to n=3).

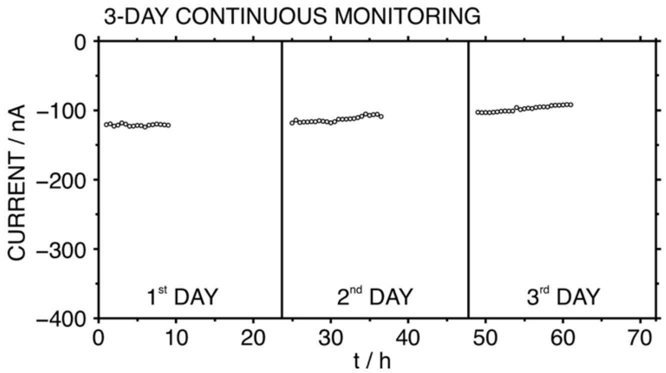

Figure S11. 3-day (72 h) continuous monitoring the 1 mM Lac in AISF with a Lac-MN sensor patch (two-electrode system). The signal was recorded every half an hour.

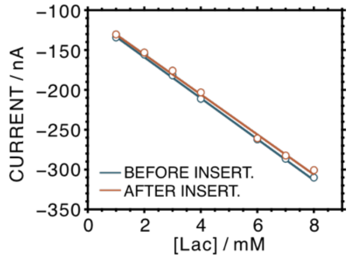

Figure S12. Calibration graphs of the Lac WE-MN before and after inserted into rat skins.

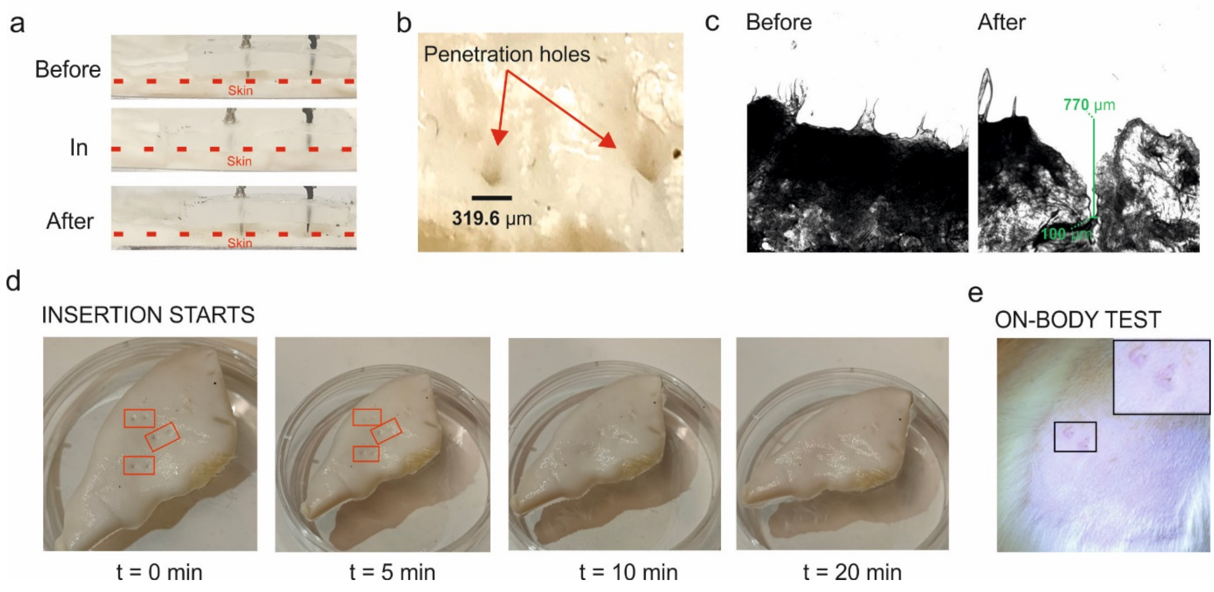

Figure S13. (**a**) Demonstration of hand-made penetration in rat skin. The depth of penetration is limited by the size of the microneedles. (**b**) Penetration holes observed after the penetration. (**c**) Histological section of a rat skin before and after microneedle insertion. (**d**) Recovery of the skin to the insertion process. Photos of the skin at times of 5, 10, and 20 minutes after the insertion. **(e)** Photos of the holes observed in the rat skin after microneedles' insertion during on-body tests on rats.

4. References

(1) Wang, Q.; Molinero-Fernandez, A.; Casanova, A.; Titulaer, J.; Campillo-Brocal, J. C.; Konradsson-Geuken, Å.; Crespo, G. A.; Cuartero, M. Intradermal Glycine Detection with a Wearable Microneedle Biosensor: The First In Vivo Assay. *Anal. Chem.* **2022**, *94* (34), 11856-11864.

(2) Molinero-Fernández, Á.; Casanova, A.; Wang, Q.; Cuartero, M.; Crespo, G. A. In Vivo Transdermal Multi-Ion Monitoring with a Potentiometric Microneedle-Based Sensor Patch. *ACS Sens.* **2022**, *8* (1), 158-166.

(3) Goud, K. Y.; Mahato, K.; Teymourian, H.; Longardner, K.; Litvan, I.; Wang, J. Wearable electrochemical microneedle sensing platform for real-time continuous interstitial fluid monitoring of apomorphine: Toward Parkinson management. *Sens. Actuators B Chem.* **2022**, *354*, 131234.

(4) Guinovart, T.; Crespo, G. A.; Rius, F. X.; Andrade, F. J. A reference electrode based on polyvinyl butyral (PVB) polymer for decentralized chemical measurements. *Anal. Chim. Acta* **2014**, *821*, 72-80.

(5) Tang, Y.-S.; Yang, T.-L.; Cheng, Y.-T.; Tsai, H.-E.; Chen, Y.-S. Real-Time Dynamic Lactate Detection in a Pipeline Using a Microsensing Needle for ICU Patient Monitoring Application. In *2023 IEEE 36th International Conference on Micro Electro Mechanical Systems (MEMS)*, 2023; IEEE: pp 425-428.

(6) Yang, H.; Jiang, X.; Zeng, Y.; Zhang, W.; Yuan, Q.; Yin, M.; Wu, G.; Li, W. A swellable bilateral microneedle patch with core-shell structure for rapid lactate analysis and early melanoma diagnosis. *Chem. Eng. J.* **2023**, *455*, 140730.
